# Emotional Availability in Samples of Mothers at High Risk for Depression and With Substance Use Disorder

**DOI:** 10.3389/fpsyg.2019.00577

**Published:** 2019-03-18

**Authors:** Alessandra Frigerio, Alessio Porreca, Alessandra Simonelli, Sarah Nazzari

**Affiliations:** ^1^Child Psychopathology Unit, Scientific Institute, IRCCS E. Medea, Bosisio Parini, Italy; ^2^Dipartimento di Psicologia dello Sviluppo e della Socializzazione, Università di Padova, Padua, Italy

**Keywords:** emotional availability, substance use disorder, depression, parenting, infancy

## Abstract

**Background:** Maternal substance use disorder (SUD) and depression have been extensively associated with dysfunctions in parent-child interactions. However, few studies have compared caregiving behaviors of these mothers. The current study aims to explore maternal emotional availability (EA) in mothers with maternal SUD and depressive symptoms in order to investigate whether these conditions represent a different risk gradient for early parenting.

**Methods:** Mother-infant relationship was investigated in 18 mothers with SUD, 11 mothers at risk for depression, and 39 mothers from general population. The dyads were videotaped during a free-play session and the quality of parent-child interactions was assessed using the EA Scales (EAS) and the Emotional Attachment and EA Clinical Screener (EA2-CS).

**Results:** Mothers with SUD scored lower on sensitivity, non-intrusiveness, and non-hostility with respect to the low-risk sample, whereas mothers at risk for depression scored lower on sensitivity and non-hostility compared to the latter. No significant differences between mothers with SUD and mothers at risk for depression emerged on the EAS, whereas different specific classifications on the EA Clinical Screener were found for the SUD (i.e., Complicated), depression (i.e., Detached), and low-risk (i.e., Emotionally Available) samples.

**Conclusion:** If the current findings are replicated, they might have significant implications for selecting targets of early mother-infant interventions.

## Introduction

Maternal substance use disorder (SUD) and depression constitute severe risk conditions not only for women’s health, but also for the wellbeing of their children (e.g., Johnson et al., 1990; Hans and Jeremy, 2001; Parolin and Simonelli, 2016). In fact, there is strong evidence that both disorders have a significant impact on different domains of child development, from infancy to adolescence. Many studies have shown that children of mothers affected by SUD, in pregnancy and postpartum, are at high risk for altered physical growth (e.g., Shankaran et al., 2004), neurobehavioral and cognitive development (e.g., Moe and Slinning, 2001; Lester et al., 2002), as well as socio-emotional wellbeing (e.g., Walthall et al., 2008; Irner, 2012). Similarly, maternal depression during the perinatal period has been associated with several negative outcomes in offspring, including bio-behavioral, socio-emotional, and cognitive difficulties (for a review see Gentile, 2017; Netsi et al., 2018). Consequently, the attention of scholars is now focused on those genetic, antenatal, and postnatal environmental factors and mechanisms which may explain the association between these clinical conditions and children’s negative outcomes. Extensive animal and human evidence supports the fundamental role of a sensitive and responsive caregiver as one of the most important environmental factor influencing early infants’ development (Tottenham, 2018). Thus, in the last decades considerable attention has been directed toward the role played by dysfunctional parenting behavior in substance addicted and depressed mothers which might, ultimately, explain some of the negative outcomes in offspring.

Parental substance abuse exerts a specific detrimental effect on caregiving, affecting the reward and stress circuits responsible for the neurobehavioral networks of parenting (Rutherford et al., 2011). Other factors associated with SUD, such as the presence of psychopathological comorbidities, including primary and substance induced major depressive disorder, primary and substance induced psychotic disorder, borderline personality disorder, antisocial personality disorder (Jones and McCance-Katz, 2019), low socioeconomic level, and educational backgrounds (Bays, 1990; Zuckerman and Brown, 1993; Brooks et al., 1994; Hans, 1999; De Palo et al., 2014), as well as drug-related impairments in neuropsychological functioning (Håkansson et al., 2018; Porreca et al., 2018), contribute to make caregiving behaviors of these mothers less effective. As a consequence, mothers with SUD are described as less sensitive and responsive toward their infants’ communicative signals, as well as less likely to show contingent responsiveness and dyadic reciprocity during emotional exchanges with them (Swanson et al., 2000; Eiden, 2001; Salo et al., 2009, 2010; Flykt et al., 2012; Porreca et al., 2016). At the same time, their parenting attitudes are characterized by less positive emotional expression and more hostile behaviors both in play and feeding contexts (Fitzgerald et al., 1990; Pajulo et al., 2001; Johnson et al., 2002; LaGasse et al., 2003; Fraser et al., 2010). Moreover, as compared to mothers without SUD, mothers with SUD are usually reported as more intrusive, directive, and interfering with children’s activities during interactions, in early infancy, preschool, and school age (Bauman and Dougherty, 1983; Bauman and Levine, 1986; Rodning et al., 1991).

Similarly, specific features of affective, behavioral, cognitive and neurobiological functioning related to maternal depression are supposed to compromise the quality of caregiving, especially when other risk factors (e.g., co-occurring anxiety disorder, marital discord, and financial stress), often associated with this disorder, are present. As compared to non-depressed mothers, those with a diagnosis of clinical depression have been reported to be less involved in vocal and visual communication with them (Field, 2010), to be less sensitive and responsive to their cues (Easterbrooks et al., 2000; Trapolini et al., 2008; Vliegen et al., 2009) as well as to be less able to structure the interaction with them (Vliegen et al., 2009). Moreover, depressed mothers were found to be more likely to behave intrusively (Vliegen et al., 2009) and, although to a lesser extent, to be hostile when interacting with their children (Lovejoy et al., 2000). Similarly, most studies have found a relation between depressive symptoms and dysfunctional maternal caregiving in normative samples (e.g., Lovejoy et al., 2000; Lock and MacMahon, 2006; Riva Crugnola et al., 2016; Ierardi et al., 2018), though not all (e.g., Kim et al., 2012). Interestingly, maternal depression in infancy was also found to predict later maternal behavior, in terms of structuring and sensitivity, when children were 7 years (Easterbrooks et al., 2000) in a poverty sample, thus revealing the long term impact of depressive symptomatology on dysfunctional patterns of caregiving.

Many recent studies investigating caregiving behaviors, respectively, in samples of SUD (e.g., Salo et al., 2009, 2010; Fraser et al., 2010; Flykt et al., 2012; Porreca et al., 2016) and depressed mothers (e.g., Easterbrooks et al., 2000; Lock and MacMahon, 2006; Trapolini et al., 2008; Vliegen et al., 2009) refer to the construct of emotional availability (EA), which indicates “*the capacity of a dyad to share an emotional connection and to enjoy a mutually fulfilling and healthy relationship*” (Biringen and Easterbrook, 2012), and is operationalized via the EA Scales (EAS; Biringen, 2008). These studies focused on caregiving behaviors toward infants (Vliegen et al., 2009; Salo et al., 2010; Flykt et al., 2012; Porreca et al., 2016), toddlers (Lock and MacMahon, 2006; Salo et al., 2009) and children (Easterbrooks et al., 2000; Trapolini et al., 2008), with dysfunctional parenting of these mothers reported in all periods. However, only a handful of studies have evaluated caregiving behaviors through the EAS by the first 3 months of child’s life, despite this time frame represents a critical window because of unrelenting parenting demands on one side, and a less ability of infants to engage the caregiver in positive interactions on the other side. In particular, Fraser et al. (2010) reported lower maternal sensitivity and overall EA toward their 3-month-old infants in a small sample of low-income drug abusing mothers, as compared to mothers not at risk of substance abuse, while no differences on the others EA dimensions were found. Differently, van Doesum et al. (2007) failed to report an association between levels of depressive symptoms and maternal sensitivity, as measured through the EAS, in a sample of depressed mothers with infants ranging from 1 to 12 month-old, although the lack of a group of non-depressed mothers did not allow to directly compare the EA scores.

Moreover, to our knowledge, only Salo et al. (2010) have compared caregiving behavior, as assessed by the EAS, in 15 opioid-abusing, 15 depressed and 57 non-exposed Finnish mothers, focusing on the main EA domains in the latter half of the first year of their infant. Specifically, the authors found that opioid-abusing mothers had lower scores on sensitivity, structuring and non-intrusiveness EA dimensions in comparison with both depressed and non-exposed mothers, while there were no differences related to caregiving behavior between depressed and non-exposed mothers, with the exception of maternal sensitivity. Last, to the best of our knowledge, no studies to date have employed the Emotional Attachment and EA Clinical Screener (EA2-CS), which represents a useful measure of attachment, in drug abusing and/or depressed mothers.

The current study aims to extend previous findings from early infancy and from Salo et al. (2010) by exploring the quality of early mother-infant relationship in low-risk, SUD and at risk for post-partum depression samples of women and their 2-months-old infants through the main EAS and subscales, as well as groups classifications on the EA2-CS. We hypothesized that maternal behaviors of SUD mothers would be more impaired in comparison to those of low-risk and at risk for post-partum depression mothers. Moreover, differences in all EA measures between at risk for post-partum depression and low-risk mothers would also be expected.

## Materials and Methods

### Participants and Procedure

The total sample consisted of 68 Italian mother-infant dyads, including 18 women with SUD (SUD sample), 11 women at high risk for post-partum depression (depression sample), and 39 women recruited from general population (low-risk sample), and their 2 months-old infants (mean age = 2.26, *SD* = 0.56 months) who were mostly (91.2%) first born across the three groups.

The SUD sample consisted of mothers (mean age = 28.56; *SD* = 5.39; range = 22–41 years) placed in a therapeutic community (TC), due to a guardianship order issued by the Youth Court. They were recruited at the beginning of a rehabilitative program, which includes pharmacological, educational and psychological intervention as described in Stocco et al. (2012). Most of them presented an early onset (*M* = 16.22; *SD* = 2.56) and a pattern of poly-drug use (*N* = 15; 83.3%), with heroin as primary substance of use (*N* = 13; 72.2%), followed by cocaine (*N* = 2; 11.1%), alcohol (*N* = 2; 11.1%), and other drugs (*N* = 1; 5.6%). Mothers from the depression and the low-risk samples were part of a larger sample recruited during pregnancy for the effects of depression on infants (EDI) study, a longitudinal investigation into the effects of antenatal maternal depression on children bio-behavioral development (Nazzari et al., 2019). Specifically, the “depression sample” consisted of mothers (mean age = 31.54; *SD* = 2.42; range = 28–36 years) scoring equal or above 10, after 2 months from delivery, on the Edinburgh Postnatal Depression Scale (EPDS; Cox et al., 1987), a widely used 10-item self-report for the assessment of depressive symptoms. This cut-off has been recommended for the validated Italian version and allows to identify mild depression cases (internal consistency = 0.79, sensitivity = 0.61, and specificity = 0.95; Benvenuti et al., 1999).

The low-risk sample consisted of mothers (mean age = 29.77; *SD* = 2.01; range = 25–34 years) scoring below the EPDS cut-off when their child was 2 months of age and with no history of depression and substance abuse as assessed by the Structured Clinical Interview for DSM-IV Axis I Disorders (SCID-I; First et al., 2002).

Mothers from the three samples did not differ significantly with respect to age levels (below vs. above 30 years) [*χ*^2^(2) = 3.53; *p* = 0.17], but they did with respect to maternal education [*χ*^2^(2) = 18.87; *p* < 0.001]. Specifically, participants with less than 10 years of education were more likely to belong to the SUD sample, whereas those with more than 10 years of education were more likely to belong to the low-risk sample. Mother-child interaction was assessed during a 15-min free-play situation, which took place in the TC for the SUD sample and in the Scientific Institute Eugenio Medea for the depression and low-risk samples. The study was approved by the Ethics Committees of the Scientific Institute Eugenio Medea and the University of Padova and all mothers signed an informed written consent to participate.

### Emotional Availability Scales

The interaction was video-recorded and coded according to the parental dimensions of the fourth version of the Emotional Availability Scales (EAS; Biringen, 2008), which allow to assess four dimensions of maternal EA, namely, *Sensitivity, Structuring, Non-intrusiveness*, and *Non-hostility*. Specifically, the *Sensitivity Scale* measures the caregiver’s ability to be warm and emotionally connected with the child; the *Structuring Scale* assesses the caregiver’s ability to scaffold the child’s activities and set appropriate limits; the *Non-intrusiveness Scale* evaluates the caregiver’s ability to be available to the child without being intrusive, while the *Non-hostility Scale* measures the degree of overt and covert hostility toward the child. Each scale can be rated globally on a direct score, ranging from 1 (“less optimal” feature) to 7 (“optimal” feature). Scores between 5.5 and 7 are considered adequate and index of a healthy relationship, scores around 4 indicate inconsistency (i.e., behaviors that are appropriate in some way but that are not fully healthy), while scores of 3 or below refer to less optimal interactions where problematic behaviors can be observed.

Moreover, each scale can be rated also according to seven subscales which represent the main features of each EA dimension, with a total score ranging from 7 to 29. In particular, the first two subscales for each EA dimension are rated on a 7-point scale, while the remaining 5 subscales are rated on a 3-point scale, with higher ratings referring to more optimal features observed. As the scores obtained in the first two subscales are more strongly weighted and drive the final global score, in order to limit multiple testing, only the first two subscales for each EA dimension were considered in the subsequent analysis. Specifically, for the *Sensitivity* scale, *Affect*, which refers to the appropriateness and authenticity of the caregiver’s affect, and *Clarity of perception and appropriate adult responsiveness*, which refers to the caregiver’s ability to be responsive, were included. For the *Structuring* scale, *Provision of guidance*, which refers to the adult’s ability to appropriately structure the child’s play, and *Success of attempts*, which refers to the child receiving and responding appropriately to the adult’s attempts were selected. For the *Non-intrusiveness* scale, *Follows the child’s lead*, which refers to the adult’s ability to follow the child’s lead, and *Non-interruptive ports of entry into the interaction*, which refers to the caregiver not abruptly or physically interrupting the interaction, were included. For the *Non-hostility* scale, *Lack negativity in face or voice*, which refers to no observable evidence of negative emotionality, and *Lack of mocking, ridiculing, or other disrespectful statement*, which refers to the lack of ridiculing or mocking words or behaviors toward the child, were included.

Finally, the EA coding system allows to globally measure caregiver-child EA through the Emotional Attachment and EA Clinical Screener (EA2-CS; Biringen, 2008; Saunders et al., 2015), which is scored on a 100-point scale divided in 4 EA zones conceptually mapped onto the attachment categories: Emotionally Available (81–100), Complicated (61–80), Detached (41–60), and Problematic (1–40). Specifically, caregivers who present a warm, healthy, and secure emotional connection with their children (i.e., that are optimally sensitive and responsive to their signals) are rated as Emotionally Available; those who present emotional warmth within a less healthy relationship, usually characterized by dependency and over-connection (i.e., that are inconsistently sensitive), are rated as Complicated; those who are not warm, but rather mechanical and emotionally flat (i.e., insensitive to child’s cues) are rated as Detached; last, caregivers who are either traumatizing or traumatized, or who present more extreme relational features, are rated as Problematic.

Therefore, the EA coding system enables to move through different levels in the evaluation of parent-child relationships, allowing to describe more global patterns of EA (the EA zones measured through the EA2-CS), to consider each EA dimension (the EAS), or to focus more specifically on the basic components of each dimension (the subscales).

Two independent and certified reliable coders (AP and SN) rated the videos. The inter-rater agreement was calculated on a subsample of 24 cases, randomly selected from each sample, through intra-class correlations which showed values ranging from 0.66 to 0.96 (mean = 0.81) for the EAS, and Cohen’s Kappa equal to 0.80 for the EA2-CS.

### Statistical Analyses

Chi-square analyses were conducted to examine possibly confounding group differences related to maternal sociodemographic characteristics (i.e., age levels and education). Separate one-way multivariate analyses of variance (MANOVA) were used to investigate group (SUD, depression and low-risk samples) differences in both EAS and subscales, while LSD *post hoc* tests were conducted to explore all possible pair-wise comparisons of means. Last, in order to analyze possible associations between the three groups and the EA2-CS classifications, chi-square analysis was performed and adjusted standardized residuals examined.

## Results

Preliminary analyses showed no effect of maternal age and education on the 4 EAS scores (all *p*’s > 0.05), thus both variables were not included as covariates in the main analyses. Descriptive data for the EAS in the three samples are reported in Table 1.

**Table 1:** Descriptive statistics for the EA scales in the three samples.

		EA SCALES							
		
		Sensitivity		Structuring		Non-intrusiveness		Non-hostility	
					
	*N*	*Mean*	*SD*	*Mean*	*SD*	*Mean*	*SD*	*Mean*	*SD*
**Maternal age**									
Below 30 years	30	4.60	1.18	4.58	0.93	4.75	1.32	6.05	0.66
Above 30 years	38	4.79	1.15	4.87	0.98	5.01	1.10	6.08	0.68
**Maternal education**									
Less than 10 years	14	4.61	0.79	4.68	0.89	4.46	1.05	5.82	0.50
More than 10 years	54	4.73	1.24	4.76	0.98	5.01	1.22	6.13	0.69
**Samples**									
SUD	18	4.17	0.91	4.39	0.99	4.11	1.21	5.64	0.48
Depression	11	4.18	1.25	4.54	0.99	4.68	1.33	5.82	0.75
Low-risk	39	5.06	1.15	4.96	0.90	5.26	1.07	6.31	0.63


There was a statistically significant difference in EA scores based on groups [*F*(8, 124) = 2.51, *p* = 0.01; Wilk’s Λ = 0.740]. One-way MANOVA showed significant differences among the mean scores of the three samples on the Sensitivity [*F*(2, 65) = 5.39; *p* = 0.007; partial η^2^ = 0.14], Non-intrusiveness [*F*(2,65) = 6.27; *p* = 0.003; partial η^2^ = 0.16], and Non-hostility [*F*(2,65) = 8.14; *p* = 0.001; partial η^2^ = 0.20] scales. *Post hoc* comparisons using the LSD test revealed that mothers from the SUD sample scored lower on Sensitivity, Non-intrusiveness, and Non-hostility scales (all *p*’s < 0.01) than mothers from the low-risk sample. Mothers from the depression sample scored lower than mothers from the low-risk sample on the Sensitivity and Non-hostility scales (all *p*’s < 0.05).

Further, there were statistically significant differences in EA Sensitivity subscales [*F*(4, 128) = 2.69, *p* = 0.03; Wilk’s Λ = 0.851], Structuring subscales [*F*(4, 128) = 2.35, *p* = 0.05; Wilk’s Λ = 0.868], Non-intrusiveness subscales [*F*(4, 128) = 4.90, *p* = 0.001; Wilk’s Λ = 0.752] and Non-hostility subscales [*F*(4, 128) = 3.06, *p* = 0.02; Wilk’s Λ = 0.833] based on groups: the mean scores of the three samples differed according to all the main two subscales included in each of the four EAS, with the exception of the “Provision of Guidance” subscale (see Table 2). However, *post hoc* comparisons on the EA subscales scores showed that mothers from the depression and the SUD samples scored lower than mothers from the low-risk sample only on the two subscales of the Sensitivity dimension (*p*’s < 0.05), while, for all the remaining comparisons, differences on the EA subscales were only found between the SUD and the low-risk samples (all *p*’s < 0.05).

**Table 2:** Descriptive statistics and comparisons for the EA subscales in the three samples.

EA subscales	Low-risk	Depression	SUD			
				
	Mean (SD)	Mean (SD)	Mean (SD)	*F*	*p*	partial η^2^
**Sensitivity**						
Affect	5.08 (1.18)	4.23 (1.46)	4.08 (0.90)	5.46	0.006	0.14
Clarity of perceptions and appropriate responsiveness	4.92 (1.03)	4.23 (1.23)	4.08 (0.83)	5.04	0.009	0.13
**Structuring**						
Provision of guidance	4.99 (0.90)	4.68 (0.90)	4.42 (1.00)	2.41	0.098	0.07
Success of attempts	4.79 (0.96)	4.18 (1.03)	4.14 (0.89)	3.73	0.029	0.10
**Non-intrusiveness**						
Follows child’s lead	5.29 (1.10)	4.73 (1.27)	3.94 (1.08)	8.92	0.000	0.22
Non-interruptive ports of entry into interaction	5.08 (1.10)	4.68 (1.47)	4.08 (0.99)	4.69	0.012	0.13
**Non-hostility**						
Lack negativity in face or voice	6.23 (0.71)	5.82 (0.81)	5.56 (0.62)	6.07	0.004	0.16
Lack of mocking, ridiculing or other disrespectful statement and/or behavior	6.18 (0.72)	5.73 (0.85)	5.67 (0.64)	3.85	0.026	0.11


Last, as shown in Table 3, a significant association between groups (SUD, depression, and low-risk samples) and EA2-CS (Emotionally Available, Complicated, and Detached) classifications emerged [*χ*^2^(4) = 16.06; *p* = 0.003]. Specifically, mothers from the low-risk sample were more likely to be classified as Emotionally Available (adjusted standardized residuals = 3.2), those from the depression sample as Detached (adjusted standardized residuals = 2.6), and those from the SUD sample as Complicated (adjusted standardized residuals = 2.5). None of the mothers was classified as Problematic.

**Table 3:** Distribution of the EA2-CS classification in the three samples.

EA-zones	SUD	Depression	Low-risk
	
	N (%)	N (%)	N (%)
Emotionally Available	2 (11.1)	2 (18.2)	20 (51.3)
Complicated	13 (72.2)	4 (36.4)	15 (38.5)
Detached	3 (16.7)	5 (45.4)	4 (10.3)
Problematic	0 (0)	0 (0)	0 (0)


## Discussion

The current study is among the first to investigate EA in mothers from two samples (SUD and at risk for post-partum depression) at high risk for disturbances in early parenting in comparison with mothers from a low-risk sample as early as 2 months after birth. Specifically, we focused on different levels of analysis in the evaluation of maternal behaviors according to the EA method, ranging from the four “classical” dimensions (i.e., “Sensitivity,” “Structuring,” “Non-intrusiveness,” and “Non-hostility” scales) and their main components (i.e., subscales) to a more global measure of attachment (i.e., EA2-CS classification).

In line with our expectations, mothers with SUD showed impairments in almost all EA domains in comparison with mothers from the low-risk sample, thus extending results of previous studies evaluating maternal EA on mothers-infant dyads at older ages, which reported impaired sensitivity (e.g., Salo et al., 2009, 2010; Fraser et al., 2010), high intrusiveness (e.g., Salo et al., 2010) and hostile behaviors (e.g., Salo et al., 2009) in this population. These findings were also reflected by significantly lower scores on the main subscales of the EA dimensions than those obtained by mothers from the low-risk sample. Specifically, mothers with SUD presented less optimal affects and difficulties in correctly perceiving and responding to their infants’ signals, as well as troubles in following the child’s lead and waiting for optimal ports of entry into interaction. Moreover, they showed more negativity in face or voice and disrespectful statements or behaviors toward their infants. Interestingly, while mothers with SUD did not obtain lower scores on the Structuring scale, in line with the results of other studies (Salo et al., 2009), significant differences on a specific subscale of this EA domain (i.e., Success of Attempts) were also found. In particular, these mothers showed precise difficulties in offering bids that could be successful in scaffolding the infant’s abilities and in moving him/her toward a zone of proximal development. In the light of the emphasis on the dyadic quality of this subscale, we might speculate that this finding might be, at least partly, influenced by specific characteristics of infants prenatally exposed to substances. There is evidence that these infants show difficulties in attention, arousal, and affect regulation (Tronick and Beeghly, 1999; Frank et al., 2001), which in turn might prevent them from adequately “receiving” parental structuring. Moreover, it might be that these processes are exacerbated by less functional parental structuring bids, further compromising the success of attempts (Lester and Tronick, 1994), although this remains an open question for future studies.

Similarly, according to the four EA dimensions, mothers at risk for post-partum depression were more likely to be less sensitive and more hostile toward their 2 months-old infants than mothers from the low-risk sample, in line with previous evidence (e.g., Lock and MacMahon, 2006; Trapolini et al., 2008). However, it is important to mention that a handful of studies failed to detect an association between maternal depressive symptoms and EA in both normative (Kim et al., 2012) and clinically depressed (van Doesum et al., 2007) samples, although the lack of comparison samples in those studies might partially explain the null finding. Moreover, only the scores on the two subscales (“Affect” and “Clarity of Perception and Appropriate Responsiveness”) related to the dimension of sensitivity were found to be significantly lower in at risk for post-partum depression mothers in comparison with controls, while no differences between the means of the two groups on the Non-hostility subscales emerged. It can be speculated that while the condition of SUD exerts a significant impact on both the global and fine grained components of negative emotion regulation, the effect of depressive symptoms on this domain can be detectable only on a more global level of clinical observation. In other words, it might be hypothesized that several behaviors (including those captured by the five remaining Non-hostility subscales, not specifically examined in the current analyses) related to overt and covert hostility may be present in women at risk for postpartum depression, although at lower intensity levels, and globally contribute to the overall score of the main scale.

No significant differences were found between SUD and at risk for post-partum depression mothers on the EAS as well as on the main subscales of the EA dimensions, in contrast to what previously reported by Salo et al. (2010). It can be supposed that methodological differences, related to sample’s characteristics and infants’ age range, account for this contradictory result. Specifically, in the Finnish study, opioid abusing mothers received a replacement therapy during pregnancy and were offered various forms of early support after birth (i.e., pediatric consultations, professional residential or outpatient treatments focusing on promoting mother-child relationship), although not all of them were able or willing to participate. In contrast, SUD mothers from the current sample were all resident in a TC and were all involved in a mandatory therapeutic program involving an integrated daily treatment. It can be speculated that participants from the Salo et al. (2010) study were at higher risk for dysfunctional parenting than ours, as also reflected by the lower mean scores on the EAS in their sample as compared to the current one. At the same time, at risk for post-partum depression Finnish mothers were beginning an intervention program in a clinic specialized in the treatment and evaluation of early relationship problems, while mothers at risk for post-partum depression from our sample were not receiving any intervention. Moreover, the focus on older infants (i.e., 7–9-month-olds) in Salo et al. (2010) study, might further explain inconsistencies with the current findings.

Importantly, we first reported specific patterns of EA, as measured through the EA2-CS classification, from each group. Specifically, Emotionally Available, Complicated, and Detached classifications were more represented among low-risk, SUD and at risk for post-partum depression mothers, respectively. Thus, it can be speculated that the EA2-C2, that is thought to reflect the dyadic emotionally-based attachment of a caregiver-child relationship (Biringen, 2008), is able to distinguish a risk gradient for parenting related to different clinical conditions. While it is expected that mothers from low-risk samples behave in a sensitive manner with their infants, it is noteworthy that over 70% of SUD mothers were classified as Complicated, while only 17% as Detached and none of them as Problematic. Although a complicated classification refers to a non-optimal mother-child relationship, “Complicated” mothers are generally warm and kind with their child, as well as responsive at times. Specifically, these relationships are usually characterized by the concurrent presence of emotional warmth and less functional aspects, such as inconsistency or incoherence, dependency, and over-connection. Thus, it can be hypothesized that SUD mothers are somehow able to create an emotional connection with their children, despite showing consistent difficulties in sensitivity, non-intrusiveness and non-hostility as highlighted by the EAS. We might speculate that the TC may act as a protective or deterrent factor against the manifestation of more severe forms of emotional unavailability. Specifically, the coercive nature of involuntary community treatment might have partly pushed mothers to “perform” better. In contrast, almost half of women with depressive symptoms showed very minimal emotional connection with their infants, resulting mechanical and distant when interacting with them (i.e., Detached), with potential negative implications for the development of infants’ responsiveness and a healthy mother-child relationship, as suggested by the association between maternal depression and insecure attachment found in several studies (reviewed in Wan and Green, 2009). Despite the current findings are preliminary, they seem to suggest that global measures of mother-child relationship, as that provided by the EA2-CS, might be able to capture distinct dyadic pattern of mother-child relationship in samples at high risk for dysfunctional parenting as early as 2 months after birth, thus highlighting the potential of the EA2-CS classification as a useful research and clinical tool. Future studies should investigate the developmental sequelae of such complicated and detached early relationship on child later outcomes.

Globally, the findings of the present study seem to suggest a different impact of clinical conditions related to SUD and risk for post-partum depression on maternal EA, ranging from impairments in single EA dimensions to effects that are more likely to affect the overall quality of mother-child relationship, determining different patterns of EA, thus encouraging the adoption of different levels of observation in the investigation of parent-child relationships. Moreover, another strength of the current study is represented by the specific focus on caregiving behaviors in a particularly sensitive period (i.e., early infancy) of child’s life, during which the impact of risk for maternal post-partum depression and SUD could be particular detrimental with respect to child development and to the establishment of early mother-child interactive patterns (Cohn and Tronick, 1983; Lester et al., 2002; Tronick et al., 2005). Nevertheless, it is essential to emphasize that the cross-sectional and observational nature of the current study does not allow us to draw any causal inferences. Additionally, it is likely that child characteristics, such as negative emotionality, which is often found in infants of SUD (Behnke et al., 2013) or depressed mothers (Shapiro et al., 2018), might interact with maternal risk factors to affect maternal EA (e.g., Cecil et al., 2017) and contribute to explain the current findings.

Some limitations of our study are noteworthy and have to be considered. First, the lack of diagnosis of depression and the small number of women in the at risk for postpartum depression group limit the generalizability of our findings to clinical populations and restrict the power of statistical analyses to detect significant differences. Second, the heterogeneity of the SUD sample jointly with, once again, its small sample size, prevented us to investigate the impact of specific characteristics related to this disorder (e.g., type and duration of substance use) on parenting. Additionally, we cannot exclude that the presence of comorbid depressive symptoms and/or personality disorders in the SUD sample might concur to explain the impact of drug addiction on caregiving behaviors. Furthermore, women from the SUD sample were more likely to have a lower education status in comparison with women from the low-risk sample. Third, our results concern maternal dysfunctional caregiving behaviors, associated with SUD and the risk for depression, acted toward 2 months-old infants and cannot therefore be applied to similar populations of mothers with children of a different age range. Further studies with larger samples are needed to confirm our preliminary data and generalize our findings to clinical populations of depressed mothers, different samples of SUD, and different infants’ ages.

To sum up, our results show that mothers with SUD and mothers at risk for post-partum depression are less sensitive, more intrusive (just for the former) and more hostile with their 2 months-old infants than mothers from the low-risk sample, as also reflected by specific “ingredients” of the EA dimensions. Moreover, they also suggest that both risk groups might present distinctive patterns of impaired EA with significant implications for selecting targets of early mother-infant interventions.

## Data Availability

The datasets generated for this study are available on request to the corresponding author.

## Author Contributions

AF and AS prepared the study design and supervised the research team. AF wrote the manuscript with input from all authors and performed the statistical analysis. AP and SN recruited the samples and rated all videos. All authors discussed the results and contributed to the final version of the manuscript.

## Conflict of Interest Statement

The authors declare that the research was conducted in the absence of any commercial or financial relationships that could be construed as a potential conflict of interest.
